# Neurodegeneration in Cognitive Impairment and Mood Disorders for Experimental, Clinical and Translational Neuropsychiatry

**DOI:** 10.3390/biomedicines12030574

**Published:** 2024-03-05

**Authors:** Simone Battaglia, Alessio Avenanti, László Vécsei, Masaru Tanaka

**Affiliations:** 1 Center for Studies and Research in Cognitive Neuroscience, Department of Psychology “Renzo Canestrari”, Cesena Campus, Alma Mater Studiorum Università di Bologna, 47521 Cesena, Italy; 2 Department of Psychology, University of Turin, 10124 Turin, Italy; alessio.avenanti@unibo.it; 3 Neuropsicology and Cognitive Neuroscience Research Center (CINPSI Neurocog), Universidad Católica del Maule, Talca 3460000, Chile; 4 Department of Neurology, Albert Szent-Györgyi Medical School, University of Szeged, Semmelweis u. 6, H-6725 Szeged, Hungary; vecsei.laszlo@med.u-szeged.hu; 5 HUN-REN-SZTE Neuroscience Research Group, Hungarian Research Network, University of Szeged (HUN-REN-SZTE), Tisza Lajos krt. 113, H-6725 Szeged, Hungary

## 1. Introduction

Neurodegeneration poses a significant challenge for the fields of neuroscience and medicine, as it is the underlying cause of the development and advancement of numerous neurodegenerative and psychiatric disorders [1,2,3]. It encompasses the progressive decay and loss of neurons across various levels of organization, ranging from molecular to network levels [4,5,6,7]. Onset can manifest at various life stages, ranging from early phases, as observed in neurodevelopmental disorders, to later stages, exemplified by conditions like Alzheimer’s disease (AD) [8,9,10]. Neurodegeneration has the potential to impact cognitive, emotional, and behavioral functions, as well as the neural mechanisms associated with consciousness and attention [11,12,13]. Hence, comprehending the mechanisms and repercussions of neurodegeneration is imperative in order to identify risk factors, biomarkers, and therapeutic targets [14,15,16]. Nevertheless, the existing therapies for neurodegenerative disorders primarily address alleviate symptoms but are largely inadequate in terms of efficacy. Hence, there is a requirement for new and inventive methods, such as non-invasive brain stimulation, that can regulate neural activity and plasticity in a secure and reversible manner [17,18,19,20,21]. The field is rapidly evolving, with a focus on identifying new avenues of clinical research, elucidating potential mechanisms for the therapeutic effects of non-invasive brain stimulation (NIBS) and exploring the potential synergy between different stimulation protocols and pharmacological interventions [22,23,24,25,26,27].

The study of neurodegeneration in cognitive impairment and mood disorders is a vast and intricate domain that encounters numerous obstacles in comprehending, diagnosing, and treating these conditions [28,29,30]. Several existing obstacles include: The diverse and inconsistent nature of neurological and psychiatric disorders, posing challenges for the identification of shared mechanisms, biomarkers, and therapeutic targets across various subtypes, stages, and populations [31,32,33,34,35]. The absence of efficacious disease-altering treatments for the majority of neurodegenerative disorders, which restricts the available choices and results for patients and caregivers; The ethical and practical considerations associated with carrying out clinical trials and translational research in vulnerable and diverse populations, such as the elderly, children, and minority groups [36,37,38]. The integration and interpretation data derived from various origins and modes, including genetics, epigenetics, proteomics, metabolomics, imaging, electrophysiology, and neuropsychology [39,40,41,42,43,44,45,46,47]. The development and validation of novel methods, such as NIBS, artificial intelligence, and drug repurposing, which necessitate thorough examination and assessment of their safety, effectiveness, and mechanisms [11,48,49,50,51,52,53,54]. Addressing these challenges requires collaborative efforts among researchers, clinicians, patients, and policymakers from various fields to enhance our understanding and improve the treatment of neurodegeneration underlying cognitive impairment and mood disorders [55,56,57,58]. This special issue focuses on the most recent advancements and hurdles in this area, examining them from experimental, clinical, and translational perspectives.

## 2. Special Issue Articles

### 2.1. Stroke and Neuroprotection

Stroke is a critical health issue characterized by the interruption of blood flow to the brain, leading to the death and harm of neurons. Neuroprotection is a key strategy aimed at protecting neurons from damage and preserving their survival and function [59,60,61]. In this special issue, three articles examined various approaches to promote neuroprotection in animal models of stroke [62,63,64]. An article by Cruz-Martínez Y et al. investigated the impact of symbiotic supplementation, comprising probiotics and prebiotics, on memory and neuronal survival in rats suffering from ischemic stroke [62]. This study tested the effects of a symbiotic (inulin and Enterococcus faecium). The symbiotic reduced inflammation, protected neurons, and improved memory in subacute phase. This suggests that symbiotics may be useful for stroke treatment and prevention.

To lower the risk of lacunar stroke, a type of stroke that affects the brain’s small blood vessels, Zhang L et al. examined the viability of drug repurposing, a strategy that involves using fully approved drugs to treat different medical conditions. The authors used a two-sample Mendelian randomization analysis estimating the genetic variant-exposure and the genetic variant-outcome associations to identify which drugs can prevent lacunar stroke, a type of cerebral infarction [63]. This study found that genetic variants that mimic the effects of calcium channel blockers, statins, ezetimibe, and antisense anti-apoC3 agents can reduce the risk of the condition. The study suggests that these drugs should be repurposed for lacunar stroke prevention to promote healthier brain aging.

The third article by Baliellas et al. examined the impact of propentofylline (PROP), a xanthine derivative, on strengthening antioxidant defenses and decreasing lipid peroxidation in the brainstem of rats with gliotoxic injury, which serves as a model for neurodegeneration [64]. The authors tested the effects of PROP, a drug that reduces inflammation in brain cells in rats exposed to a toxic substance that causes oxidative damage in the brain. The study found that PROP prevented an increase in lipid peroxidation, a marker of oxidative stress, and enhanced the activity of glutathione reductase, an enzyme that recycles antioxidants, in the rat brainstem. This study concluded that PROP could protect the brain from oxidative damage and neurodegeneration. These articles offer a new and valuable understanding of the mechanisms and advantages of neuroprotection against stroke and related disorders.

### 2.2. Cognitions in Schizophrenia (SCZ), Multiple Sclerosis (MS), and Down Syndrome (DS)

Schizophrenia (SCZ) is a complex mental disorder that affects a range of cognitive capabilities, including memory, attention, reasoning, and language [65,66,67]. Treatment for this condition generally involves the use of antipsychotic medication, as well as psychotherapy and psychosocial interventions [68,69,70]. Nevertheless, the outcomes of these treatments can vary depending on several factors [71,72,73]. This special issue features three articles that explore various aspects of cognition in individuals with SCZ [74,75,76]. The articles explored how cognition is affected by factors such as the onset and duration of psychosis, severity of symptoms, level of dissociation, and resistance to treatment. Panov et al. examined the relationship between working memory, attention, and SCZ [74]. The study found that most patients with SCZ had problems with working memory and attention and that these problems were worse in patients who did not respond to treatment. The study also found that working memory and attention problems were linked to disorganized behavior, duration of illness, and dissociative symptoms. The study suggests that working memory and attention could be used as indicators of SCZ progression and treatment response.

In another article, de Oliveira et al. investigated the feasibility of utilizing metallic nanoparticles present in the bloodstream as biomarkers for assessing cognitive performance. The research team explored how the blood levels of metallic nanoparticles affect the cognitive abilities of people with multiple sclerosis (MS) [75]. This study measured the blood levels of eight different metals and two cognitive tests in 21 patients with MS. The authors found that higher blood levels of iron, zinc, and total metals were associated with better cognitive performance. This study proposed that blood iron concentration could be a useful indicator of cognitive impairment in people with MS. 

Furthermore, they examined the application of artificial intelligence as a means of improving the diagnosis and treatment of SCZ and related conditions. Koul et al. presented a review of Down syndrome (DS), a genetic disorder that causes intellectual and physical impairments [76]. This work discusses how artificial intelligence and machine learning can help diagnose and treat DS by analyzing various data sources. The text highlights the benefits of these technologies in understanding and improving the lives of people with DS. Overall, these articles provide novel knowledge that contributes to our understanding of the cognitive impairments and difficulties experienced by individuals with SCZ and their caregivers.

### 2.3. Depression and Antidepressants

Major depressive disorder (MDD) is a widespread and debilitating mood disorder that impacts a substantial number of individuals globally [67,77,78,79]. Characterized by persistent feelings of sadness, reduced interest, diminished self-worth, and various physical and mental symptoms, it often co-occurs with other conditions, such as anxiety, chronic pain, and neurodegenerative diseases [80,81,82,83]. In this special issue, three articles explored different approaches for diagnosing and treating depression and its comorbidities [84,85,86]. The first article assessed the neuroprotective and swiftly acting antidepressant-like properties of 20 essential oils in mice. Tran et al. conducted a study aimed at assessing the potential of essential oils as rapid-acting antidepressants [84]. The study utilized cell and animal models to evaluate the neuroprotective, anti-inflammatory, and behavioral effects of essential oils. The results indicated that certain essential oils and their constituents, possibly operating through glutamate receptors, exhibited positive effects on these parameters. The study recommended additional research on Atractylodes lancea and Chrysanthemum morifolium essential oils. 

In the second article, Cui et al. suggests that stimulated parotid saliva is a more accurate indicator of depressive disorder than unstimulated saliva. The authors conducted a study to investigate the influence of various saliva collection methods on cortisol levels, which are thought to be indicative of this emotional state [85]. The results of the study revealed that unstimulated whole-saliva cortisol was most closely related to blood cortisol levels, while stimulated parotid salivary cortisol was the most reliable predictor of the negative emotional condition. Furthermore, the study confirmed that individuals with depression had higher salivary cortisol levels compared to healthy controls, and that salivary cortisol levels demonstrated a positive correlation with the severity of the condition. The study proposed that salivary cortisol could serve as a useful non-invasive method for monitoring MDD. 

In the third article, Rajkumar examines biomarkers associated with neurodegeneration in post-traumatic stress disorder (PTSD), a condition that has the potential to initiate or exacerbate depressive symptom. The author conducted a comprehensive review of the relationship between PTSD and neurodegenerative diseases, including AD and Parkinson’s disease [86]. According to the review, a range of biomarkers, such as brain structure, genetics, inflammation, metabolism, and sleep, are linked to both PTSD and neurodegenerative disorders. The review also delved into the potential mechanisms and implications of these associations. The review found that PTSD may contribute to an increased risk of developing neurodegenerative diseases and recommended preventive measures.

### 2.4. Drug Repurposing and Cancer

Cancer is a diverse array of diseases that is distinguished by the uncontrolled expansion and invasion of abnormal cells into neighboring tissues [79,87,88,89]. The treatment of cancer often involves surgical intervention, chemotherapy, radiation therapy, and immunotherapy; however, these approaches have limitations and may produce adverse effects [90,91,92]. Therefore, the process of repurposing existing drugs for new applications, referred to as drug repurposing, offers a promising strategy for the development of novel and potent anticancer agents or adjuvants [58,93,94,95]. In this special issue, three articles were published that explored the potential of drug repurposing in the context of cancer and its associated challenges [96,97,98]. One article by Moura et al. assessed the anticancer properties of atorvastatin, a medication used to reduce cholesterol levels, and nitrofurantoin, an antibiotic [96]. The authors tested the efficacy of repurposed drugs on breast cancer and neuroblastoma cells to determine their effectiveness, both individually and in combination with doxorubicin. The results indicated that both drugs decreased the viability of both cell lines, and the combination of atorvastatin and nitrofurantoin was more effective in SH-SY5Y cells than in MCF-7 cells. The study underscores the potential use of these drugs in treating breast cancer and neuroblastoma.

In another study, Olasehinde et al. examined the beneficial impact of apigenin, a flavonoid present in plants, on mitigating cognitive and neurobehavioral impairment caused by chemotherapy. The authors conducted a comprehensive review of studies that investigated the effects of apigenin, a plant compound, on various aspects of memory and behavior in animal models of neurological disorders [97]. The review found that apigenin exhibited cognitive and neurobehavioral enhancing effects and modulated several molecular and biochemical pathways related to neuroprotection. However, the review also emphasized the need for further research to establish the optimal dosage and duration of apigenin treatment and to evaluate its efficacy in human subjects. 

In the third article, Stojsavljević et al. investigated the correlation between mercury exposure and autism spectrum disorder, a neurodevelopmental condition that may elevate the likelihood of developing cancer [98]. The authors carried out a meta-analysis of studies that investigated mercury levels in various biological samples of children with and without autism. This study revealed that children with autism exhibited higher blood, plasma, and red blood cell mercury levels, but not in their hair and urine. The review proposed that children with autism had impaired mercury detoxification and excretion and that exposure to mercury could exacerbate their condition. Furthermore, the study recommended decreasing Hg^++^ exposure and closely monitoring Hg^++^ levels in children with autism. These articles offer new perspectives on the mechanisms and applications of repurposed drugs in cancer research and therapy.

## 3. Conclusions

This special issue showcases a series of papers that delve into the theme of neuroprotection from diverse perspectives and disciplines. These papers cover a wide range of conditions that affect the brain and nervous system, such as stroke, MS, DS, MDD, PTSD, breast cancer, and neuroblastoma. Additionally, the studies examine the potential of various agents and strategies to enhance neuroprotection, including symbiotics, drugs, essential oils, salivary cortisol, apigenin, and repurposed drugs. These studies have revealed the intricate and multifaceted mechanisms and pathways that underlie neuroprotection, including oxidative stress, inflammation, neurotransmission, neurogenesis, and epigenetics [99,100,101,102,103]. The papers also emphasize the importance of non-invasive and personalized approaches for monitoring and improving neuroprotection, such as blood iron concentration, artificial intelligence, and mercury levels. The application of these new techniques, advanced real-time analysis algorithms, machine learning, and physiological biomarkers may streamline the mental healthcare process, alleviating the social burden and economic pressures commonly associated with psychiatric disorders [104,105,106,107]. These papers make significant contributions to the field of neuroprotection by advancing knowledge and practice, and suggest new avenues for future research and intervention. The special issue highlights the importance and relevance of neuroprotection in preventing and treating various neurological disorders, and promoting brain health and well-being.

## Data Availability

Data sharing is not applicable to this article.

## Abbreviations

AD	Alzheimer’s disease
DS	Down syndrome
MDD	major depressive disorder
MS	multiple sclerosis
NIBS	non-invasive brain stimulation
PROP	propentofylline
PTSD	post-traumatic stress disorder
SCZ	schizophrenia
